# Clinical evidence that the pandemic from 1889 to 1891 commonly called the Russian flu might have been an earlier coronavirus pandemic

**DOI:** 10.1111/1751-7915.13889

**Published:** 2021-07-13

**Authors:** Harald Brüssow, Lutz Brüssow

**Affiliations:** ^1^ Department of Biosystems Laboratory of Gene Technology KU Leuven Leuven Belgium; ^2^ Internal Medicine, Angiology and Gastroenterology Specialist Neuss Germany

## Abstract

Contemporary medical reports from Britain and Germany on patients suffering from a pandemic infection between 1889 and 1891, which was historically referred to as the Russian flu, share a number of characteristics with COVID‐19. Most notable are aspects of multisystem affections comprising respiratory, gastrointestinal and neurological symptoms including loss of taste and smell perception; a protracted recovery resembling long covid and pathology observations of thrombosis in multiple organs, inflammation and rheumatic affections. As in COVID‐19 and unlike in influenza, mortality was seen in elderly subjects while children were only weakly affected. Contemporary reports noted trans‐species infection between pet animals or horses and humans, which would concur with a cross‐infection by a broad host range bovine coronavirus dated by molecular clock arguments to an about 1890 cross‐species infection event.

## Abstract 2

Contemporary clinical reports on patients of the Russian flu pandemic of 1889 to 1891 show striking similarity to COVID‐19 patients, demonstrating *multiorgan affections of the respiratory, intestinal and neural system, *loss of smell and taste, *and long disease symptom duration.

Are we at a turning point of the COVID‐19 pandemic, with case numbers decreasing in countries where vaccination is increasing, or have we still not reached the peak of the pandemic yet? How many infection waves will we still see and what will the future of the SARS‐CoV‐2 virus be? Will it disappear or become endemic? It is difficult to give answers to these questions. Mathematical models provide some predictions, but some basic parameters are still so poorly defined or constrained by epidemiological data making predictions rather uncertain. Therefore, one might be tempted to take historical pandemics as paradigms to provide us with past experience and a framework for possible outcomes of the COVID‐19 pandemic. Instead of predictions, this approach could provide insights from "retrodictions". The COVID‐19 pandemic is quite unique and has been called a once in a lifetime medical emergency. Comparison with the SARS epidemic from 2002 to 2003 is therefore of limited help. Due to specific traits of the SARS‐CoV infection – particularly the onset of viral excretion after symptom onset – this epidemic was successfully controlled by public health measures which led to the eradication of the virus. Despite being caused by a related coronavirus, such an outcome is unlikely for SARS‐CoV‐2. The MERS coronavirus infection showed an even more limited spread than SARS. One might therefore be tempted to compare COVID‐19 with the Spanish flu pandemic from 1918 to 1919, but this epidemic was caused by a very different pathogen, influenza virus H1N1, and claimed an estimated 50 million lives. The COVID‐19 human tally stands in mid‐May 2021 at 3.3 million notified deaths, but, when based on estimates of excess mortality worldwide, extra fatality might come close to 10 million deaths, approaching the dimension of the Spanish flu pandemic. However, drawing inferences from a distinct viral infection might be misleading since influenza viruses and coronaviruses differ too much in biological properties. In the present Lilliput, we explore experiences from the ‘Russian flu’ pandemic of 1889 to 1891 as a possible comparator to COVID‐19. The ‘Russian flu’ pandemic claimed the lives of an estimated 1 million humans from a world population of 1.5 billion people and represents thus one of the great epidemics of the 19th century (Valleron *et al*., 2010).

## Was the Russian flu caused by an influenza virus?

As the name implies, the Russian flu was described as influenza. However, at the time viruses were still unknown and in 1892, Richard Pfeiffer, a collaborator of Robert Koch, isolated a bacterium, which he called *Haemophilus influenzae*, postulating – erroneously – to be the etiological agent of the Russian flu pandemic. Since the oldest influenza viruses were isolated and kept as laboratory stocks only since the 1930s, direct evidence for linking influenza viruses with the Russian flu is lacking. In contrast, a direct virological proof for the attribution of the Spanish flu from 1918 to 1919 to an influenza virus has been achieved by a heroic effort combining detective work finding pathological samples and corpses of pandemic victims buried in permafrost soils, followed by a technological ‘tour de force’ to reconstitute and literally revive this pandemic influenza virus in the laboratory. Nothing comparable has been achieved for the Russian flu virus of the 1889 pandemic. The attribution of this pandemic to an influenza virus relies on indirect, albeit quite interesting evidence. A Dutch virologist established the argument that the presence of haemagglutination‐inhibiting (HI) antibodies against a human or animal influenza virus in the sera of persons of a certain age‐group suggest that a related virus circulated in man. The transition from seropositivity in older age groups to seronegativity in younger age groups provides an indicator when the virus in question circulated in the human population. To illustrate this point: he investigated sera collected in the Netherlands from human subjects for antibodies against a range of influenza viruses. 70% of sera collected in 1956 from persons aged 68 years or older contained HI antibody against the Hong Kong H3N2 strain, which a decade later caused the 1968 flu pandemic. In contrast, only 10% of younger blood donors showed such antibodies (Masurel, 1969). Researchers from Harvard University and the Centers for Disease Control confirmed and extended these observations with data from US citizens. They also observed that during the 1968 to 1969 Hong Kong influenza virus pandemic, people born before 1890 showed a lower age‐specific attack rate of 5.8% compared to 15% in people born after 1895. As a negative control, no such difference was seen in the two birth groups during the 1966 influenza epidemic (18.9% vs. 20.4%, respectively) (Schoenbaum *et al*., 1976). Taken together, these data support an interpretation that people born before 1890 had antibodies cross‐reacting with influenza virus A/Hong Kong/68 and were therefore protected against infection: the prevalence of such antibodies was 90% in the cohort born before 1890, but only 5% in the cohort born after 1899. When people born before 1890 were vaccinated with the Hong Kong strain, they showed a 3‐ to 4‐fold higher pre‐immune and post‐vaccination titres to influenza virus A/Hong Kong/68 than people born after 1899. A possible interpretation is that people born before 1890, but not those born after 1899 were exposed to an influenza virus sharing the H3 antigen with the A/Hong Kong/68 strain. A literature review presented at a 1999 WHO conference on influenza surveillance concurred with this interpretation (Dowdle, 1999). Similar ‘sero‐archaeological’ approaches conducted with sera drawn in 1967 showed a sudden increase of antibody prevalence directed against the H1 antigen from a swine influenza virus to 80% in subjects born in 1918 or before when compared with subjects born after 1926, who showed only a 10% prevalence for these antibodies. The serological conclusion that the Spanish flu was caused by an H1 virus was later confirmed by the ‘resurrection’ of the H1N1 virus, giving strong support to the sero‐archaeological approach for reconstituting influenza epidemics indirectly by serological means. However, the discussion does not seem to be settled since other researchers still search historical serum collections to confirm or refute these conclusions (Altschuler *et al*., 2009).

## A coronavirus candidate for the Russian flu?

Fifteen years ago, the discussion of the agent responsible for the Russian flu pandemic of 1889 took a new turn with an unexpected observation from virologists working at KU Leuven in Belgium. They sequenced the human coronavirus OC43 (HCoV‐OC43), a group 2 or Betacoronavirus like SARS‐CoV and SARS‐CoV‐2, which caused SARS and COVID‐19, respectively. Within the Betacoronaviruses, HCoV‐OC43 belongs to the Embecovirus Lineage A, while SARS‐CoV and SARS‐CoV‐2 belong to the Sarbeco Lineage B coronaviruses. This classification difference also reflects a clinical difference: HCoV‐OC43 causes mild upper respiratory tract infections and only rarely severe pneumonia in neonates and aged people with underlying illnesses. Together with human coronavirus HCoV‐229E, a group 1 or Alphacoronavirus, it causes up to 30% of seasonal cold infections (Killerby *et al*., 2018). According to serological studies, infections with these two coronaviruses occur frequently in young children and then repeatedly throughout life. Neutralizing antibodies to these coronaviruses are found in in 50% of school‐age children and 80% of adults (Pohl‐Koppe *et al*., 1995). KU Leuven scientists then showed that HCoV‐OC43 shared very high nucleotide sequence identity with bovine coronavirus (BCoV) across the entire genome length, ranging from 93.5% for the S (spike) gene to 98% for the E (minor envelope) gene. The bovine coronavirus was shown to be the closest relative of HCoV‐OC43, except for gene E which showed 99.6% nucleotide (nt) identity with porcine hemagglutinating encephalomyelitis virus (PHEV), suggesting a potential recombination event with another coronavirus. In addition, HCoV‐OC43 shows a 290‐nt deletion affecting two nonstructural genes from BCoV. The Belgian virologists suggested that this animal‐human zoonotic pair of coronaviruses should be analysed in order to gain insights into the processes of adaptation of a nonhuman coronavirus to a human host, which is important for understanding the interspecies transmission events that might lead to the origin of human epidemics. Notably, with an estimated 4.3 × 10^‐4^ substitutions per site per year, the time to the most recent common ancestor of HCoV‐OC43 and BCoV was dated by three methods to 1891, 1873 and 1890 (Vijgen *et al*., 2005). Recent re‐examination of the molecular clock data led to a narrower estimate around 1890. Since it is not unusual to use molecular dating to investigate the origin of viral epidemics, HCoV‐OC43 has been suggested as another candidate for the 1889 to 1891 Russian flu pandemic, proposing a potential bovine trans‐species infection. Sequencing of the porcine PHEV for example revealed a rather distinct S gene compared to BCoV and HCoV‐OC43, suggesting deviation from a common ancestor already around 1780 (Vijgen *et al*., 2006). The conclusion of an 1889 to 1891 coronavirus pandemic should – even more than the alternative H3 influenza virus association – be interpreted with caution since both arguments are derived from indirect data, namely serological or molecular dating. In addition, a temporal association is not yet a proof of causality. In the following, we will investigate whether clinical and epidemiological observations allow inferences about the Russian flu pandemic to indicate a more likely influenza or coronavirus aetiology.

## Bovine coronavirus transspecies infections

Precedence of potential trans‐species infections between cattle and humans have been reported. Virologists isolated Human Enteric Coronavirus (HECV) HECV‐4408 from diarrhoea fluid of a 6‐year‐old child with acute diarrhoea (Zhang *et al*., 1994). Coronaviruses are occasional electron microscopic stool findings in humans with gastroenteritis, but have not been considered as important causes of gastroenteritis in humans. Interestingly, serological and molecular analysis showed a relationship between HECV‐4408 with virulent bovine coronaviruses and nt sequence sharing up to 99% over the HE gene, which led the authors to hypothesize that these viruses may infect and cause disease in human subjects and calves alike. Indeed, when US veterinarians orally inoculated gnotobiotic calves with HECV‐4408, all calves developed diarrhoea lasting 5–9 days. When these calves were then challenged with bovine coronavirus, no diarrhoea or virus shedding was detected while control calves developed diarrhoea and faecal and nasal virus shedding. This observation demonstrates cross‐infection and cross‐protection between humans and calves with shared coronaviruses (Han *et al*., 2006). A trans‐species infection of humans by a bovine coronavirus that resulted in the 1889 pandemic is thus not a far‐fetched hypothesis.

This argument also applies to other human coronaviruses. Four human CoVs (HCoV‐HKU1, HCoV‐229E, HCoV‐NL63 and HCoV‐OC43) are globally endemic, causing mild to moderate respiratory tract disease. Serological, molecular, receptor and cell culture infection data suggest that the endemic human virus HCoV‐229E, an alphacoronavirus, may constitute an ancient descendant of camelid‐associated viruses (Corman *et al*., 2016).

## 19th century cattle epidemics

The Belgian virologists supported their hypothesis of a bovine coronavirus causing the Russian flu pandemic with arguments that highly infectious respiratory disease with a high mortality rate affected cattle herds around the world in the 19th century, leading to massive culling operations in the period between 1870 and 1890 thus exposing many humans to large amounts of bovine viruses (Vijgen *et al*., 2005). 19th century veterinary records indeed speak of catastrophic cattle epidemics leading to importation bans of US or Russian cattle into Britain. The socioeconomical conditions of the epoch were conducive for epidemics (Hall, 1965). The British human population increased from 11 million in 1801 to 21 million in 1851 accompanied by a population shift from the countryside into towns and cities. Feeding the exploding population thus became a problem. Dairy activities were still locally organized in the countryside at small scale facilities, refrigeration was marginal and transport was slow before the build‐up of a railway network. To address these problems, live animals and – as historical reports document visibly diseased cattle – were being sold in the Metropolitan markets. In this way, many people in densely populated areas came into close contact with bovine pathogens increasing the chances of trans‐species infections. Britain suffered a series of introductions of cattle diseases: in 1839, cattle imported from the European continent brought foot‐and‐mouth disease, a picornavirus infection. In 1841, contagious bovine pleuropneumonia (CBPP) was imported. The disease is caused by mycoplasma (*Mycoplasma mycoides*) and well known since the 18th century. It was spread from Britain with the colonial expansion to the US, Australia and South Africa. It is characterized by a variable clinical picture ranging from nearly asymptomatic infections to outbreaks with up to 70% mortality, but the lung and chest pathology is very characteristic. A national disaster followed in Britain in 1865 with the introduction of the rinderpest virus, a measles‐like paramyxovirus. It spread from cattle imported from the Baltic via seaports and railway transport and could only be contained once slaughter of all diseased animals became obligatory and payment for the lost animals was introduced. In the United States, Texas cattle fever caused by a protozoon (*Babesia bigemina*) transmitted by ticks was a major cattle epidemic at this time period. It is today difficult to deduce the aetiology of specific 19th century cattle epidemics and thus difficult to assess whether bovine coronaviruses figured among the agents circulating ahead of the 1889 Russian flu pandemic. A relatively detailed contemporary 40‐page veterinary report of the rinderpest epidemic written in 1865 refers to the epidemic still as ‘murrain’, an umbrella term of epidemic diseases of cattle and sheep used since medieval times, which does not differentiate between distinct veterinary infections (Mishra, 2011). In fact without laboratory tests, even a contemporary veterinary doctor cannot diagnose a bovine coronavirus infection only based on clinical symptoms, since too many pathogens cause similar diseases.

## Bovine coronavirus infections

Further insight into this question whether the Russian flu was caused by a coronavirus, particularly a bovine coronavirus, can be expected from current veterinary experience with BCoV infections (Franzo *et al.,* 2020; Saif and Jung, 2020; Colina *et al*., 2021; Hodnik *et al*., 2020). Two features characterize BCoV: first, it has a broad host range including wild ruminants and a substantial zoonotic potential and second, it has a dual tropism for the respiratory and gastrointestinal tracts. BCoV‐like viruses, all belonging to the Embecovirus subgenus of the Betacoronavirus genus, cause gastroenteritis in sheep, goat, llama, dromedary camel, while in alpaca BCoV‐like viruses cause both gastroenteritis and respiratory infections. BCoV is shed in both feces and upper respiratory tract secretions and is endemic in cattle worldwide. BCoV is associated with different pathologies in the bovine species. One manifestation is ‘Calf diarrhoea’ causing severe, malabsorptive diarrhoea persisting for 2–8 days in newborn (< 3 weeks old) calves. It infects epithelia of the intestine leading to villous atrophy and crypt hyperplasia. Nasal shedding of virus is observed but respiratory affection is variable. Older calves of 2–6 months of age also suffer from respiratory infections with BCoV associated with coughing and rhinitis, and occasionally with pneumonia. Virus shedding is from nose and pharynx. Nasal shedding can be long (3 weeks) and recurrent and not all shedding episodes are symptomatic. Asymptomatically, virus‐shedding calves can experimentally infect seronegative calves thus potentially serving as virus reservoirs in the field. ‘Winter dysentery’ affects adult dairy and beef cattle with a haemorrhagic diarrhoea associated with respiratory signs, apathy, anorexia and fever and decreased milk production in dairy cows. Older animals are more severely affected. Morbidity is high, but of short duration (1–6 days) and mortality is low (1–2%). The pathology consists of colonic crypt necrosis. Another manifestation of BCoV is the ‘Bovine Respiratory Disease complex’ (BRDC) displaying rhinitis, pneumonia, diarrhoea, fever, anorexia and decreased weight gain. Pathologically villous atrophy is observed in the jejunum and ileum, and emphysemas and necrosis in the lung. It typically affects 6 to 10 month‐old cattle arriving after transport (‘shipping fever’) in feedlots. BRDC is a ‘complex’ infection: BCoV seems to initiate the disease, but viral co‐infections with bovine respiratory syncytial virus, parainfluenza‐3 virus or bovine herpesvirus and the outgrowth of commensal bacteria of the nasal cavity (*Mannheimia haemolytica, Pasteurella* sp., *Mycoplasma*) induced by transport stress and transmitted by intermingling with new animals in crowded feedlots contribute to the disease.

BCoV has a wide host range creating potential virus reservoirs. BCoV‐like viruses were isolated from captive wild ruminants (several deer species, elk, giraffe). Faecal viruses from a waterbuck of a wildlife farm could infect gnotobiotic calves, where it caused profuse diarrhoea and the coronavirus antigen was detected in both the alimentary and the respiratory tract of the experimentally infected calves. Two wild deer species showed serum antibodies to these BCoV‐like viruses with a prevalence of 7% (Tsunemitsu *et al*., 1995). Cross‐infection is not limited to ruminants: closely related coronaviruses were also isolated from dogs with respiratory disease. Canine infectious respiratory disease is a highly contagious disease in rehoming centres or training kennels, where it causes mild cough. Coronavirus was detected in 20% of the respiratory tract samples of dogs by RT‐PCR and the isolated virus showed 97% nt sequence identity over the S gene with BCoV and HCoV‐OC43, but was distinct from canine enteric coronavirus. Notably, an enteric BCoV could experimentally infect 1 month‐old dogs, leading to no disease, but BCoV was detected in oral and rectal swabs by RT‐PCR and induced seroconversion to the bovine virus. Control pups kept with the inoculated pups were also infected, demonstrating the high transmissibility of BCoV by asymptomatic animals (Kaneshima *et al*., 2007). The host range extends even to birds: BCoV inoculated into 1‐day old turkey poults induced an enteritis and reduced growth. Coronavirus was detected in the intestine by immune electron microscopy using anti‐BCoV antibodies and the birds seroconverted to BCoV antigen (Ismael *et al*., 2001).

## Clinical symptoms in Russian flu patients

We now come to the critical question whether the clinical symptoms reported for the Russian flu patients better fit an influenza virus infection or a trans‐species infection with a bovine coronavirus or another infectious agent. To address this question, we are in the privileged position to have two comprehensive contemporary reports from Britain and Germany on the Russian flu pandemic. The British Parsons Report (see below) raised this point in discussing alternative agents such as dengue as potential agent, but rejected this possibility.

### The British Parsons Report

In 1891 a 344‐page ‘Report on the Influenza Epidemic of 1889–90 by Dr. Parsons with an Introduction by the Medical Officer of the Local Government Board’ appeared in London, summarizing the worldwide epidemiological data for the pandemic (Report on the Influenza Epidemic of 1889‐90 ‐ Great Britain. Local Government Board, Henry Franklin Parsons ‐ Google Books; Further Report and Papers on Epidemic Influenza, 1889‐92: With an … ‐ Great Britain. Local Government Board ‐ Google Books). It also presents data on the symptoms observed in patients from different institutions in England.

*Hospitalreports:* Dr Low from St Thomas’s Hospital, London, wrote: ‘The invasion is sudden; …with acute pains in the back … often accompanied by vertigo and nausea, and sometimes actual vomiting of bilious matter. There are pains in the limbs and general sense of aching all over; frontal headache of special severity; pains in the eyeballs, increased by the slightest movement of the eyes; shivering; general feeling of misery and weakness, and great depression of spirits, … weeping; nervous restlessness; inability to sleep, and occasionally delirium. In some cases catarrhal symptoms are observed… eyes are injected; sneezing and sore throat; and epistaxis, swelling of the parotid and submaxillary glands, tonsilitis, and spitting of bright blood from the pharynx may occur. There is a hard, dry cough of a paroxysmal kind, worst at night. …There is often tenderness of the spleen. The temperature is high at the onset (100° F. in mild cases to 105° F in severe cases).’

Another physician noted: ‘The chief symptoms are coldness along the back, with shivering… severe pain in the head and eyes, …; pains in the limbs, … even in the fingers and toes; and febrile temperature, which may in the early period rise to 104° or 105° F. The patient feels excessively ill and prostrate, is apt to suffer from nausea or sickness and diarrhoea, and is for the most part restless, though often drowsy….the patient may recover in the course of three or four days. He may even have it so mildly that, although feeling very ill, he is able to go about his ordinary work. … patients have additionally some dryness or soreness of the throat, or some discharge from the nose, … accompanied by slight bleeding. … at a time when the patient seems to be convalescent, he begins to suffer from wheezing in the chest, cough, and perhaps a little shortness of breath, and before long spits mucus … tinged with blood…. Another complication is diarrhoea. Another is a roseolous spotty rash….’.

The Parsons report continues: ‘the sudden onset, rapid development of fever, and great and enduring nervous prostration is out of all proportion to the severity of the other general or local symptoms.’ It emphasizes ‘the small mortality from the disease’, but notes at the same time ‘the liability to relapses and dangerous pulmonary sequels’. ‘Catarrhal symptoms have been less prominent which led some observers to doubt whether the recent epidemic has been one of true Influenza’. Then: ‘the most common and urgent symptoms being the frontal headache and pain in the eyeballs, muscular pains in various parts of the body, and nervous depression’. ‘A rash, not unlike that of German measles, was seen in some cases, principally on the posterior aspect of the limbs’.

*Reportsfrom prison and asylum:* Dr. Cowan, medical officer to the Pentonville Prison, is quoted in the report with: ‘Period of incubation: from one to seven days. General aspect: The patient looks ill and has a dull drowsy appearance. Prostration: Very marked, and a general desire to go to bed. Headache: Very severe, commencing at back of neck, and settling down into a severe frontal headache with post‐orbital pains. Temperature averages 101°F, which only lasts about 24 h. Pain: Especially in head, back, and thighs. Sore throat noticed in a few cases. Diarrhoea in a few cases. Complications: Bronchitis with pain over sternum, but very little sputum. A dry hacking irritable cough, lasting about five days.’

The Parson report then continues with observations from 70 infected adult patients in an asylum of the insane in Edinburgh, published by the British Medical Journal on February 1st, 1890. The major symptoms were: great weakness (92%), frontal headache (88%), pain in limbs (84%), giddiness (81%), loss of appetite (78%), coryza (nasal discharge associated with common cold) (77%), bronchitis (77%), nausea (62%). Gastrointestinal signs were less frequent e.g. vomiting (38%) and diarrhoea (25%).

*Observationsresembling COVID‐19:* A number of observations described in the Parsons report resemble more characteristics of COVID‐19 than those of influenza. Notable are:

*Light affection in adolescents:* ‘Among 177 cases in a girl’s school reported in the British Medical Journal of February 22nd, 1890 headache (98%), watery eyes (96%) and flushed face (80%) were the major symptoms. Among 85 adolescent boys frontal headache was the only symptom observed in more than 50% of the cases.’

*Children are relatively spared:* in the words of the Parsons report ‘It was by many considered that children were not so liable to contract Influenza as adults, but the large per‐centage affected in some schools and training ships negatives this view. It seems, however, generally agreed that children who contracted Influenza did not have it so severely as adults, suffering less pain and being sooner convalescent.’

*Age as risk factor for mortality:* ‘Influenza was a disease especially fatal to elderly persons’.

*Comorbidity as risk factor for mortality:* ‘An attack of Influenza greatly tends to bring about or hasten a fatal termination if occurring in a patient who is already the subject of organic disease of the heart, phthisis pulmonalis (today: pulmonary tuberculosis), or pulmonary emphysema; and also, according to the statistics of Dr. Bertillon, diabetes or cerebral disease. It is also especially dangerous to persons advanced in life.’

*Gender bias for morbidity:* ‘Some medical men stated that more males suffered than females.’

*Long haulers:* ‘The long enduring evil effects of an access of Influenza in a large proportion of cases suggests that the *materies morbi* is only slowly extinguished in or eliminated from the system. Some subjects experience a weekly attack or relapse for many weeks after the primary access. It may take the form of great impairment of mental and physical power, or the more definite shape of vertigo or cardiac depression with general arterial relaxation necessitating recourse to the recumbent position.… Relapses …are of frequent occurrence; they occurred in 9% of the cases.’

*Pathology:* ‘the local phenomena may be the result of minute thromboses in the different organs of the body’ and ‘of the complications the most frequent are inflammatory conditions of the respiratory organs, as pneumonia, bronchitis, and pleurisy, and to these the mortality ascribed to it is chiefly due.’

*Multisystem disease:* ‘By many observers three forms of Influenza have been recognized, viz.: A. Nervous, B. Catarrhal, C. Gastric. These three forms have all been observed in cases occurring together under the same roof, and are evidently mere varieties of the same disease.’

*Presymptomatic transmission:* ‘It has been suggested by a German observer that the patient may be capable of communicating infection, while as yet only in the stage of incubation. If so, this would help to explain the rapid spread of the disease.’

*Occasional symptomatic reinfection:* ‘A case is recorded in the British Medical Journal of February 15th, 1890, in which a patient who had suffered from Influenza in France in December 1889, had another attack in England in January 1890.’

*Lack of immune protection from previous influenza epidemic:* ‘The persons now living who passed through the (influenza) disease in 1847 are of course comparatively few, but such persons have not been exempt from the present epidemic.’

### The British Medical Journal reports

The 1889 pandemic was well covered in contemporary reports published in the British Medical Journal (Kousoulis and Tsoucalas, 2017). Eade (1891) reported on cases he treated in East Anglia. Some characteristics resemble more COVID‐19 than classical influenza. He observed multiorgan affection ranging from the respiratory system (catarrh, dry spasmodic asthma, bronchitis) over gastrointestinal symptoms (nausea, vomiting, diarrhoea) to marked neurological symptoms. The latter comprised mental disturbances, dulled conditions of the brain, apathy and affections of sensory nerves. Skin affections were observed and included alopecia (loss of hairs). Pulmonary inflammation was the most frequent cause of death and affected the very old and the previously diseased. He noted frequent and severe nervous sequelae in cases from 1890. Regions severely affected in 1890 were nearly spared in 1891 suggesting a single agent and the development of herd immunity. When reporting on Influenza occurring in 1893–1894, the same British physician described a disease that corresponded to classical influenza with mortality peaks both in the very young and the very old persons and nearly exclusive lung affection with few other symptoms and no neurological sequels highlighting the different nature of the two diseases (Eade, 1894). Another British report reinforced the resemblance of the 1889–1892 epidemic with COVID‐19 when noting that ‘the most common sequelae found have been nerve depression, neuralgia, headaches, and loss of taste and smell’ and describing pulmonary, intestinal and rheumatic forms, frequently mixed. The physicians also observed a ‘peculiar immunity of young children’ untypical for influenza (Anonymous, 1892). A combination of respiratory, gastrointestinal and neurological symptoms were also reported in Australia for residents of a mental disease asylum, where ‘tedious convalescence was almost general’ during the pandemic wave (Hay, 1892).

### The Britannica entry

Further insight is provided by an *Encyclopaedia Britannica* entry on ‘Influenza’ published in 1911 (1911 Encyclopædia Britannica/Influenza – Wikisource, the free online library). At that time the phrase ‘Influenza simulates other diseases’ was coined (Clifford Allbutt) and it was reported that ‘cardiac attacks were common leading to the idea that a specific toxin for heart muscle was produced as well as a nervous toxin. In the Paris epidemic of 1890 the suicides increased 25%, a large proportion of the excess being attributed to nervous prostration caused by the disease.’ Dr Rawes treating in 1889–1890 influenza patients at St Luke’s hospital in London, is quoted with ‘insanities traceable to influenza melancholia is twice as frequent as all other forms of insanity put together. Other common after‐effects are neuralgia, dyspepsia, insomnia, weakness or loss of the special senses, particularly taste and smell, abdominal pains, sore throat, rheumatism and muscular weakness. The feature most dangerous to life is however the special liability of patients to inflammation of the lungs.’

### The German ‘Verein für Innere Medicin’ Report

When considering that in 1889 the germ theory of infectious diseases had not yet won over the theory of miasma, where foul air released from fissures in the soil opened up by earthquakes were argued to be at the base of epidemics, a report collected by the German association of internal medicine issued in 1892 at Berlin appears strikingly modern in its approach (Leyden and Guttmann, 1892 https://collections.nlm.nih.gov/catalog/nlm:nlmuid‐64820270R‐bk). This association designed a questionaire comprised of 15 subject areas regarding observations with patients from the Russian flu epidemic in Germany. The survey was sent to 20 000 medical doctors over all regions of Germany and 6000 returned detailed answers which were then systematically evaluated by subcommittees of this association according to symptom complexes. The 189‐page report accompanied by numerous maps can be summarized as follows (translation from the authentical text by the authors are marked by ‘…’, explicative terms in parantheses by the present authors).

*Cardiovascular observation:* ‘Affection of the heart particularly in elderly and obese subjects could be life threatening. Many patients report a feeling of constriction of the thorax and precordially localized anxiety. Pericarditis and endocarditis was repeatedly mentioned. Rheumatic disease was noted as complications. The most interesting complication affects the vascular bed because this has not previously been seen in other epidemics. Phlebitis and thrombosis was frequently observed in the recovery phase, even deadly cases of sinus thrombosis occurred. Striking were cases of thrombosis in arteries.’

*Respiratory observations:* ‘Nearly without exception coryza (rhinitis) was observed, associated with lacrimation and sneezing attacks; epistaxis (nose bleeding) was very frequent as was local swelling of lymph nodes. Pharyngitis and tonsillitis frequently led to laryngo‐tracheitis where the feeling of an irritation in the larynx caused coughing. The irritation descends from the bronchi into the smallest bronchiole. Initially there is a dry cough which later culminates in paroxysm of coughing. Dyspnea (shortness of breath) is frequently observed.’

*Pneumonia:* ‘This epidemic was not only characterized by the high frequency of pneumonia, observed in 5 to 10% of all infected subjects, but particularly by the observation of croupous pneumonia (defined by fibrinous matter in the air vesicles of the lung). The mortality in patients with pneumonia was very high and ranged from 15 to 26%. Death occurred by lung edema and heart paralysis. Bronchopneumonia started with shivering and slow temperature increases. Pneumoniae were most frequent in the elderly and persons with weak constitution. In middle‐aged people pneumonia occurred without fever. Effusion into the pleura cavity was observed in 12 to 20% of the cases. The most important complication was cerebral meningitis in the convalescence phase which was not observed in any of the previous epidemics. Sequels were reported for people with cardiac diseases and diabetes. Rare cases of hemorrhagic lung infarcts with embolic material from upper leg veins were described. Tuberculosis was exacerbated by the infection. Preterm delivery was reported.’

*Gastro‐intestinal observations*: ‘The GI tract was affected in nearly all patients, more severe gastrointestinal symptoms were however only observed in a quarter of all cases. The disturbance was manifold and could persist for 4 weeks particularly in children. In 18% of subjects only the stomach, in 15% only the intestine was affected. Loss of appetite was accompanied by strange changes in taste perception. The patients reported either complete loss of taste or abnormal taste perception describing as bitter or putrid taste impressions. Vomiting occurred in 34%, diarrhoea in 34% of the patients and 15% suffered from both vomiting and diarrhoea. Both symptoms were associated with a shorter disease course. 5% of the cases showed a haemorrhage of the intestinal mucosa.’

*Neurological observations*: ‘Neuralgic pain and prostration is prominent and for 92% of the patients neurological complaints dominated the disease. Patients noted mostly headache, and less frequent back and muscle ache. A quarter of the patients was incapable resuming their usual activity even without showing other symptoms of illness. Vertigo, sleeplessness, fainting and neuralgic pain in cranial nerves (trigeminus) were reported with a frequency ranging from 5 to 14% of the patients. Many reported substantial disturbances of smell and taste perceptions. After the acute phase, impaired memory was observed interpreted as exhaustion psychosis. General exhaustion after the infection was frequent and many patients needed several weeks to regain their former strength.’

*Skin*: Half of the patients showed a minor, quickly disappearing exanthema (skin eruption in certain viral or coccal infections). Petechia (pinpoint skin bleeding) and skin haemorrhage was observed and bleeding from the mucosa, particular the nose, was frequent.

*Multisystem disease*: The physicians noted several forms of disease manifestations during the pandemic which ‘they distinguished as (i) nervous, (ii) respiratory, (iii) gastric and (iv) rheumatic forms. They observed in Germany a geographically distinct representation of these four disease manifestations.’

## Epidemiological observations

The German report also documented epidemiological observations. ‘Prodromal signs were indisposition, headache and shivering. The incubation time was given as 2–6 days. Susceptibility to the infection differed between individuals: strong and obese persons were more severely affected than weak and thin persons. The physicians observed that childless couples, singles and families without social contact were not affected.’

The German survey also investigated environmental factors increasing or decreasing infection rates. Workers in tobacco factories were spared from the infection, which the authors of the survey attributed to the air disinfecting action of the fuming ovens maintained in these factories.

The observers of the 1890s suspected links of the Russian flu epidemic with veterinary diseases, particularly horse diseases, but the evidence is mostly anecdotal. For example, the German survey noted that servants in agriculture and in cavalry troops who slept in barns with horses were largely spared from the pandemic. This report also mentioned severe infections in horses followed by infections in the farmer’s family (case study from East Prussia) or an epidemic in cavalry horses, followed a month later by infections first in cavalry and then in infantry soldiers (case study in Bucharest). Possible human‐animal cross‐infections were also described for pet animals: a case report described an infection transmitted from infected family members to a cat which showed bronchitis, somnolence and apathy; and in another report transmission from infected family members to a dog and in another case report to a parrot where the animals showed fatigue, decreased appetite, and cough.

Also British physicians investigated zoonotic links. Eade (1891) noted an outbreak of infections in horses (‘pink eye disease – a conjunctivitis unrelated to influenza or coronavirus infections) preceding the 1891 epidemic in humans. Veterinary authorities of the time said that there is ‘no trustworthy evidence of the communication of the disease from man to horse or from horse to man’, while other veterinarians maintained that ‘some weeks before the epidemic had reached its height, the omnibus and cab service (by horses in Brighton) was completely disorganized’ (Anonymous, 1892). In a rural area near Nottingham affected by the 1889 epidemic, a BMJ paper reported that ‘horses in the neighbourhood have been affected with a cough, with profuse discharge from the nostrils, swollen submaxillary glands, and inability to work, lasting about ten days’ (Tibbles, 1890). These symptoms resemble equine influenza (Neumann *et al*., 2021) and not equine coronavirus infections, which cause rare enteric infections in horses (Pusterla *et al*., 2018). Prior to the 20th century, equine and human ‘influenza’ epidemics were often temporally associated (Neumann *et al*., 2021), but solid evidence for a zoonosis from horses to humans cannot be deduced from the historical records.

## Comparisons

It is of course difficult to formulate a hypothesis for a microbiological aetiology of a pandemic that occurred 130 years ago, at an epoch when viruses were still unknown and Koch’s postulates for a pathogen were first presented in 1890 at a scientific meeting in Berlin and where contemporary biological samples are totally lacking. Based on clinical and epidemiological observations, the disease was called by the original authors influenza, but already the authors of the Parsons report had doubts about the description as influenza and discussed dengue as an alternative diagnosis for the Russian flu pandemic from 1889 to 1891. Dengue infection is through hindsight an unlikely explanation, but differentiating an influenza virus infection from a COVID‐19 patient purely on clinical ground is even today for a physician a difficult task. The symptoms are overlapping. Uncomplicated influenza is characterized by an abrupt onset of symptoms after an incubation period of 1 to 2 days. Clinically, fever, chills, headache, myalgia, malaise and anorexia are observed. Prostration is noted in severe cases. Pain in the back muscles and eye muscles, arthralgia, dry cough, severe sore throat, nasal obstruction, substernal discomfort is frequently found (Treanor, 2005). Some of these signs were also described in historical records for patients of the Russian flu pandemic, but are now also observed in COVID‐19 patients. A major complication of influenza leading to fatality is pneumonia either as primary viral pneumonia or as secondary bacterial pneumonia from a coinfection with *Streptococcus pneumoniae*, *Haemophilus influenzae* and particularly *Staphylococcus aureus* (Treanor, 2005). COVID‐19 pneumonia is in contrast a pure viral pneumonia (Russell *et al*., 2021). For the Russian flu, some early bacteriology was done leading to the hypothesis of *H. influenzae* as infectious agent which was however later dropped from the list of the suspects. Non‐pulmonary complications of definitive influenza also include cardiac complications (myocarditis, pericarditis) and neurological complications (Guillain‐Barré syndrome, myelitis and encephalitis), but they are only rarely observed in influenza patients (Neumann *et al*., 2021). From the clinical symptoms noted for the Russian flu in the British and German reports, one gets the impression of a multiorgan disease which fits much more the clinical observations of COVID‐19 than those of influenza. Particularly the gastrointestinal symptoms – amply documented in the German survey from 1892 – appear very similar to observations from COVID‐19 patients. Indeed, SARS‐CoV‐2 as well as many veterinary coronaviruses show a dual tropism for both the respiratory and the alimentary tract. Avian influenza viruses also show a tropism for the alimentary tract and they excrete massive amounts of virus in the faeces which is similar to COVID‐19 patients, but this is not the case for human influenza virus infections (Neumann *et al*., 2021).

Then there is the peculiar observations both made during the Russian flu pandemic and now with COVID‐19 of the loss of smell and taste perception that is not caused by nasal congestion. Since anosmia and ageusia are now used as relatively reliable clinical diagnostic markers for COVID‐19 (Bénézit *et al*., 2020; Menni *et al*., 2020), and since molecular data show a tropism of SARS‐CoV‐2 for olfactory neurons (albeit recent observations point to inflammatory reactions instead of viral cytopathology causing anosmia) (de Melo *et al*., 2021), one is tempted to attribute this specific symptom seen in the Russian flu pandemic patients more to a coronavirus than to an influenza virus infection. Human influenza viruses also show some neurotropism in mice, but not in humans.

Additionally, several epidemiological observations documented in the historical records of the Russian flu pandemic point more to a COVID‐19‐like than to an influenza‐like disease. One might mention the incubation period estimated for the German cases of the Russian flu epidemic, which is closer to the incubation period of COVID‐19 than of influenza. Influenza virus has a U‐shaped age profile of clinical susceptibility where both young children and elderly are clinically affected. COVID‐19 has its main fatality in the elderly, this was also noted for the Russian flu pandemic. While the peak mortality in the Russian flu pandemic was with the elderly, substantial mortality was also seen in adults but children suffered only mild symptoms similar to the current COVID‐19 pandemic. Other epidemiological observations also hint towards COVID‐19 rather than influenza in patients from the 1889 to 1891 pandemic, namely the predilection for obese subjects and patients with comorbidity (particularly heart disease and diabetes) and – less clear, but mentioned in the historical records – a somewhat greater impact on males than females. The long recovery period mentioned in the German survey and British publications and the frequent neurological sequels mentioned in many British case reports from the 1889 pandemic and the following years of fatigue, lack of concentration, depression and anxiety also resemble what is now described as long haulers or long covid symptoms (Honigsbaum and Krishnan, 2020). Of particular note is the frequent mentioning of persistent headaches weeks and even months after the acute infection, causing deficits in intellectual activity reported after the 1889 pandemic and now after COVID‐19, while such reports are not prevalent after the Spanish flu influenza from 1918/19 (Rozen, 2020).

A final argument – less documented by the historical data, but nevertheless intriguing – is the connection with veterinary infections. Both influenza viruses and coronaviruses are veterinary pathogens and zoonosis plays an important epidemiological role in both viral infections. For influenza, this is classically along the line water fowls to pigs to humans. In comparison, coronaviruses are notorious for their broad host range and the potential for zoonosis as now also well documented for veterinary infections with SARS‐CoV‐2 in cats, hamsters and mink. In that context, the case reports from 1892 documenting infections possibly transmitted between horses and humans, some of them associated with possible cross‐protection; and infections purportedly transmitted from humans to pet animals – including a cat and a dog – resemble closely veterinary infections documented with SARS‐CoV‐2. Influenza viruses cross species barriers, but not as easily as coronaviruses. The physicians compiling the data 130 years ago were already suspecting such zoonotic links, but the lack of knowledge of viruses and their detection prevented further investigations at that time.

There are of course also some observations suggesting an influenza infection in 1889–1892, and perhaps the most striking is the sudden onset of the symptoms. Other observations such as meningitis fit neither influenza nor COVID‐19. As the Russian flu pandemic occurred in three distinct waves, it remains possible that an influenza virus pandemic preceded or followed a coronavirus pandemic. The broad overlap of influenza and COVID‐19 symptoms makes a diagnostic differentiation difficult and this applies particularly to a historical pandemic which occurred when microbiology was a new scientific branch. In view of the possibility that the Russian flu might have been a coronavirus infection as indicated by the clinical data from the reviewed historical reports, it is tempting to analyse the relatively well‐documented epidemiological literature on the Russian flu pandemic development for hints on how the COVID‐19 might develop in the next months and years.

## Funding Information

No funding information provided.

## Conflict of intereat

None declared.
